# A dark–light transition triggers expression of the floral promoter *CrFTL1* and downregulates *CONSTANS-like* genes in a short-day plant *Chenopodium rubrum*

**DOI:** 10.1093/jxb/eru073

**Published:** 2014-03-18

**Authors:** Jana Drabešová, David Cháb, Jan Kolář, Kateřina Haškovcová, Helena Štorchová

**Affiliations:** Institute of Experimental Botany, Academy of Sciences of the Czech Republic, v.v.i. Rozvojová 223, 165 02 Prague 6, Lysolaje, Czech Republic

**Keywords:** *Chenopodium rubrum*, *CONSTANS-like*, flowering, *FLOWERING LOCUS T-like*, gene expression, light sensitivity, short-day plant.

## Abstract

The proper timing of flowering is essential for the adaptation of plant species to their ever-changing environments. The central position in a complex regulatory network is occupied by the protein FT, which acts as a florigen. We found that light, following a permissive period of darkness, was essential to induce the floral promoter *CrFTL1* and to initiate flowering in seedlings of the short-day plant *Chenopodium rubrum* L. We also identified two novel *CONSTANS-like* genes in *C. rubrum* and observed their rhythmic diurnal and circadian expressions. Strong rhythmicity of expression suggested that the two genes might have been involved in the regulation of photoperiod-dependent processes, despite their inability to complement *co* mutation in *A. thaliana*. The *CrCOL1* and *CrCOL2* genes were downregulated by dark–light transition, regardless of the length of a preceding dark period. The same treatment activated the floral promoter *CrFTL1.* Light therefore affected *CrCOL* and *CrFTL1* in an opposite manner. Both *CrCOL* genes and *CrFTL1* displayed expression patterns unique among short-day plants. *Chenopodium rubrum*, the subject of classical physiological studies in the past, is emerging as a useful model for the investigation of flowering at the molecular level.

## Summary

The floral promoter *CrFTL1* is activated by light exposure in *Chenopodium rubrum*, showing peaks of expression anti-phasic to *CONSTANS-like* genes. This activation mode is unique among short-day plants.

## Introduction

Daylength, or photoperiod, is a key environmental factor responsible for flower induction particularly in plants growing in higher latitudes. The central position in a complex network regulating flowering in *Arabidopsis thaliana* is occupied by the protein FLOWERING LOCUS T (FT; reviewed by Amasino, 2010; Pin and Nilsson, 2012), which fulfils a role of florigen, a factor long predicted to be responsible for flower induction (Corbesier *et al.*, 2007; Zeevaart, 2008). *FT* gene expression is regulated by various environmental cues including daylength (Jaeger and Wigge, 2007). Information about daylength or photoperiod is mediated by the transcription factor CO (CONSTANS) (Putterill *et al.*, 1995). The CO protein is produced under long days in *A. thaliana* and activates *FT* (Suarez-Lopez *et al.*, 2001), whereas it is degraded under short days (Valverde *et al.*, 2004; Jang *et al.*, 2008).

The CO protein contains two conserved B-box zinc finger domains at its N-terminus, which may be responsible for protein–protein interactions (Ben-Naim *et al.*, 2006; Wenkel *et al.*, 2006). The CCT (CONSTANS, CONSTANS-like, TIMING OF CAB EXPRESSION1) domain is located at the C-terminus and has been implicated in DNA binding (Robson *et al.*, 2001). A large family of 17 *CO-like* (*COL*) genes exists in *A. thaliana*, divided into three subfamilies or groups according to gene structure (Griffiths *et al.*, 2003). Group I contains the genes with two B-boxes, including *CO.* Only a minority of the *AtCOL* genes is involved in the control of flowering in *A. thaliana*. Besides *CO*, *AtCOL5* was shown to induce flowering (Hassidim *et al.*, 2009). In contrast, *AtCOL3* (Datta *et al.*, 2006) and *AtCOL9* (Cheng and Wang, 2005) inhibit flowering. Some *COL* genes appear to be involved in the regulation of plant development other than flowering, e.g. *AtCOL3* directs lateral root formation and shoot branching (Datta *et al.*, 2006).

A *CO*/*FT* regulatory module is conserved among short-day plants, which flower when night is longer than a critical period. *FT* and *CO* homologues were identified in rice and termed *Hd3*a and *Hd1*, respectively (Yano *et al.*, 2001). *Hd3a*, like *FT*, activates flowering under permissive conditions, i.e. short-day in rice. *Hd1*, a *CO* homologue, fulfills dual roles: it downregulates *Hd3a* expression under long days but promotes it under short days (Yano *et al.*, 2000). The mode of action of *Hd1* is therefore partially distinct from *CO* in *A. thaliana*. A *CONSTANS* homologue participates in the regulation of development, specifically in the induction of bud dormancy in trees (Bohlenius *et al.*, 2006), grapevine (Almada *et al.*, 2009) or in tuberization in potato (Gonzalez-Schain *et al.*, 2012).

To build a complex regulatory network managing the control of flowering, plants seem to utilize similar elements: genes with a conserved structure. However, their function or position in signalling pathways may be changed according to the specific demands of particular species. A recent example was provided by Harig *et al.* (2012), who found that three of four tobacco *FT* homologues functioned like floral inhibitors, which was atypical for FT-like proteins. A similar case was described by Pin *et al.* (2010) in sugar beet. Two *FT* homologues shared similar sequences, but displayed antagonistic functions. Whereas *BvFT2* acted as a floral promoter, *BvFT1* repressed flowering.

*Chenopodium rubrum* belongs to the same family, Amaranthaceae, as sugar beet and is a short-day plant that can be induced to flower at a juvenile stage. Owing to this convenient manipulation, it became the subject of classical physiological studies of flowering (Cumming, 1967; Seidlová and Krekule, 1973).

In the present study, we confirm *CrFTL1*’s function as a floral inducer (Cháb *et al.*, 2008) by the complementation assay in *A. thaliana*. We demonstrate its upregulation by a dark–light transition regardless of circadian phase, a mode of light response very unusual among short-day plants (Kojima *et al.*, 2002; Hayama *et al.*, 2007). We also describe two novel *COL* genes, termed *CrCOL1* and *CrCOL2*. Their strong diurnal and circadian rhythmic expression suggest their possible role in the regulation of plant development, despite their failure to complement an *A. thaliana co* mutation. Both genes were downregulated by a dark–light transition at the same time when *CrFTL1* was upregulated. Therefore, *COL* and *CrFTL1* showed complementary expression levels.

## Materials and methods

### Plant material

Seedlings of *C. rubrum* ecotype 374 (Cumming, 1967) were cultivated in growth chambers at 20 °C with constant light or under various photoperiodic treatments essentially as described by Cháb *et al.* (2008). Sampling during darkness was performed under a dim green light. To estimate flower development, 15 individuals from each experimental treatment were left intact and grown under constant light until the age of 14 days. Morphological changes at the shoot apex associated with flowering were observed under a stereomicroscope.

Seeds of *A. thaliana* were stratified for 3 days at 4 °C. Seeds were then sown onto Jiffy-7 pellets (41mm diameter, manufactured by Jiffy Products International AS (Norway)). Five seeds were sown onto each pellet. Seedlings were later thinned to two average-sized individuals at the age of 10 days and then to a single plant in each pellet at the age of 17 days. Plants were cultivated in Microclima Arabidopsis Cabinet MCA1600E-6TL (Snijders Scientific, Netherlands) at 20 °C and 60% relative humidity. Light was provided by white fluorescent tubes. Daylength was 16h of light (130 μmol m^–2^ s^–1^) daily.

### Cloning of *CrCOL* genes

Degenerate primers designed by program CODEHOP (Rose *et al.*, 1998; Supplementary Table S1, available at *JXB* online) were targeted to conserved regions of the first and second exons, respectively, of the *CONSTANS* gene. Genomic DNA from *C. rubrum* was amplified with AmpliTaq Gold DNA Polymerase (Applied Biosystems, CA, USA) under the following conditions: 9min initial denaturation at 94 °C; 36 cycles 30 s at 94 °C, 40 s at 56 °C and 1.5min at 72 °C; final extension 10min at 72 °C. Resulting 1.5kb PCR fragments were cloned in pGEM-T Easy vector (Promega, WI, USA) and sequenced. Based on this sequence, specific primers CrCON138for and CrCON1523rev (Supplementary Table S1 available at *JXB* online) were developed. These were used to amplify cDNA from *C. rubrum* seedlings using *Taq* polymerase (Promega, WI, USA) and conditions: 2min at 94 °C; 35 cycles 40 s at 93 °C, 45 s at 58 °C and 2min at 72°C; final extension 5min at 72 °C. To obtain complete sequences of *CrCOL* cDNA, a modified RACE procedure (Cháb *et al.*, 2008) was employed. Briefly, cDNA was prepared from the mixture of RNA extracted from *C. rubrum* seedlings sampled at various times of day using SMART technology (Clontech, CA, USA). This was then cloned into pGEM-T Easy vector (Promega, WI, USA) and amplified with a combination of gene-specific and vector-targeted primers (Supplementary Table S1 available at *JXB* online) using PCR with Phusion high-fidelity DNA polymerase (Thermo Fisher Scientific, Finland): 30 s at 98 °C; 35 cycles for 10 s at 98 °C, 20 s at 58 °C and 45 s at 72 °C; final extension 5min at 72 °C. As Phusion polymerase generates amplicons with blunt ends, A-tailing (Knoche and Kephart, 1999) with *Taq* polymerase (Promega, WI, USA) was performed to generate PCR fragments suitable for A/T cloning into the pGEM-T Easy vector. The introns were amplified with the specifically designed primers directed to adjacent exons (Supplementary Table S1 available at *JXB* online) and sequenced. Raw sequences were edited, stored and analysed using Vector NTI Suite 9. The newly obtained sequences were deposited in GenBank under the acccession numbers EU395770 and EU395771.

### Transformation of *A. thaliana*

Full-length cDNAs of *CrFTL1*, *CrFTL2*, *CrCOL1* and *CrCOL2s* were cloned into pGEM-T Easy vectors using the primers targeted to UTRs (Supplementary Table S1 available at *JXB* online), and confirmed by sequencing. The vector carrying *CrFTL2* was digested with *Eco*RI (MBI Fermentas, Lithuania). This restriction fragment was inserted into the *Eco*RI site of pRT101 (Töpfer *et al.*, 1987) between the polyA signal and 35S promoter. The vectors bearing other genes were cut with *Not*I (MBI Fermentas, Lithuania), the resulting fragments were filled by Klenow polymerase fragment (MBI Fermentas, Lithuania), and subsequently inserted to blunt-ended pRT101. The gene casette was transferred from pRT101 to pGreenII0179 (Hellens *et al.*, 2000) using *Hin*dIII site. To generate transgenic plants, the vector with a casette was transformed into *Agrobacterium tumefaciens* strain GV3101 carrying a helper plasmid pSoup. *Arabidopsis thaliana* wild type or mutants were transformed by floral dipping (Clough and Bent, 1998). T1 progeny was selected on a medium with hygromycin. Homozygous T2 plants were identified on the basis of the screening of their progeny on the medium with hygromycin. The presence and expression of transgenes were confirmed by PCR or qRT PCR, respectively, using EF1-α as a reference in *A. thaliana* (Landa *et al.*, 2010). The average transcript levels of the transgenes were only about 2- to 8-fold lower than the EF1-α reference level.

### RNA extraction and reverse transcription

Total RNA, extracted by means of RNeasy Plant Mini Kit (Qiagen, Germany), was treated with DNase I (DNA-free; Ambion, TX, USA). One microgram of RNA and oligo dT primers (500ng) were heated for 5min at 65 °C, chilled on ice and mixed with Transcriptor buffer (Roche, Germany), 0.5 μl of Protector RNase Inhibitor (Roche, Germany), 2 μl of 10mM dNTPs and 10 units of Transcriptor Reverse Transcriptase (Roche, Germany). The first strand of cDNA was synthesized at 55 °C for 30min. RNA samples were reverse transcribed in two independent RT reactions and each cDNA was measured twice.

### qRT PCR measurement

The first strand of cDNA was diluted 10–20 times and qPCR was performed using the LightCycler 480 SYBR Green I Master (Roche) in a final volume of 10 μl with 300nM of each of the HPLC purified primers (Supplementary Table S1 available at *JXB* online), supplied by Metabion (Germany). The LightCycler LC 480 (Roche, Germany) was programmed as follows: 10min of initial denaturation at 95 °C, then 40 cycles for 10 s at 95 °C, 8 s at 58 °C, and 15 s at 72 °C. PCR efficiencies were estimated from calibration curves generated from serial dilution of cDNAs. A calibrator was used to correct for run-to-run variation. To distinguish *CrCOL* splice variants, TaqMan probes (FAM dye, TAMRA quencher) were designed by TIB Molbiol (Germany) (Supplementary Table S1 available at *JXB* online). LightCycler 480 Probes Master kit (Roche, Germany) was applied in a final volume of 10 μl with 300nM of each of primers and 50nM TaqMan probe under the following cycling conditions: 10min of initial denaturation at 95 °C, then 45 cycles for 10 s at 95 °C, 30 s at 60 °C, and 1 s at 72 °C. The specificity of each Taqman assay was verified through qPCR reactions with plasmids carrying particular *CrCOL* inserts as a template. Crossing points obtained with non-specific targets were at least 15 cycles lower than with the corresponding splice variant. The relative ratio of the target and reference gene was calculated as follows:

$$E_{\text{R}}{}^{C_{\text{pR}}}/E_{\text{T}}{}^{C_{\text{pT}}}$$ (1)

where *E*_T_/*E*_R_ represents the efficiency of target/reference amplification and *C*_pT_/*C*_pR_ represents the cycle number at target/reference detection threshold (crossing point). Expression values were normalized with *actin* (Cháb *et al.*, 2008). Its invariant expression was confirmed by direct quantification of cDNA as described by Libus and Štorchová (2006).

### Southern hybrization

Genomic DNA (1 μg) extracted according to Štorchová *et al.* (2000) was digested with restriction enzymes, electrophoresed overnight on a 0.9% agarose gel, and transferred to a positively charged membrane (Roche, Germany) by capillary blotting. A 0.5-kb fragment of the first exon of the *CrCOL1* gene was amplified and labelled with digoxigenin (DIG) using a PCR labelling kit (Roche, Germany) according to the manufacturer. The primers used to generate the probes are provided in Supplementary Table S1 (available at *JXB* online). The membranes were hybridized with non-radioactively labelled probes and visualized as described (Cháb *et al.*, 2008).

### Data analysis

We estimated the number of days to first flower bud and first flower, and the number of stem and rosette leaves at time of flowering in transgenic, mutant, and wild-type lineages of *A. thaliana*. We performed one-way ANOVA implemented in IBM SPSS Statistics. Honestly significant differences (HSD) were determined by Tukey test. Mean values and standard errors of transcript abundances from two biological and two technical replicates were calculated using Microsoft Excel.

## Results

### Overexpression of the *CrFTL* genes in *A. thaliana*

We performed a complementation assay to verify the function of the *CrFTL* genes (Cháb *et al.*, 2008). *CrFTL1* and *CrFTL2* cDNAs driven by the CaMV 35S promoter were transformed into *A. thaliana ft-2* mutant. Flowering was dramatically accelerated in the six independent homozygous lines carrying *CrFTL1* gene. They flowered when only three rosette leaves were developed, about 2 weeks following germination (Table 1). In contrast, the five independent *CrFTL2* transgene lines examined in detail (out of 10) exhibited no effect on flowering, despite high transgene expression (Table 1). Similar results were observed when *CrFTL1* and *CrFTL2* were overexpressed in wild-type individuals. We conclude that *CrFTL1* is a functional equivalent of the *FT* gene in *A. thaliana*, whereas no clue about possible function was provided by heterologous complementation in the case of *CrFTL2*.

**Table 1.** *Flowering time (mean number of rosette leaves at time of flowering) of* A. thaliana *transformed with the* C. rubrum *genes under the control of the 35S promoter*Data were collected from at least 10 homozygous T3 plants grown under long days. Asterisks denote honestly significant difference (HSD) estimated by Tukey test.

Genotype	Number of rosette leaves (range of mean values)	Number of T1 lineages
**Experiment 1**		
*ft* mutant (*ft-2*)	10.4	
*35S::CrFTL1* (*ft-2*)	2.5***–3.2***	6
*35S::CrFTL2* (*ft-2*)	8.4–12.0	5
Wild type *Ler-0*	6.9	
*35S::CrFTL1* (*Ler-0*)	2.4***–3.5***	3
*35S::CrFTL2* (*Ler-0*)	5.9–7.5	4
**Experiment 2**		
Wild type *Ler-0*	6.7	
*35S::CrCOL1* (*Ler-0*)	6.0–8.2	4
*35S::CrCOL2s* (*Ler-0*)	5.4–7.7	4
*co* mutant (*co-2*)	17.5	
*35S::CrCOL1* (*co-2*)	17.3–18.4	5
*35S::CrCOL2s* (*co-2*)	17.3–18.4	5

### Light triggers *CrFTL1* expression

**Fig. 1.**
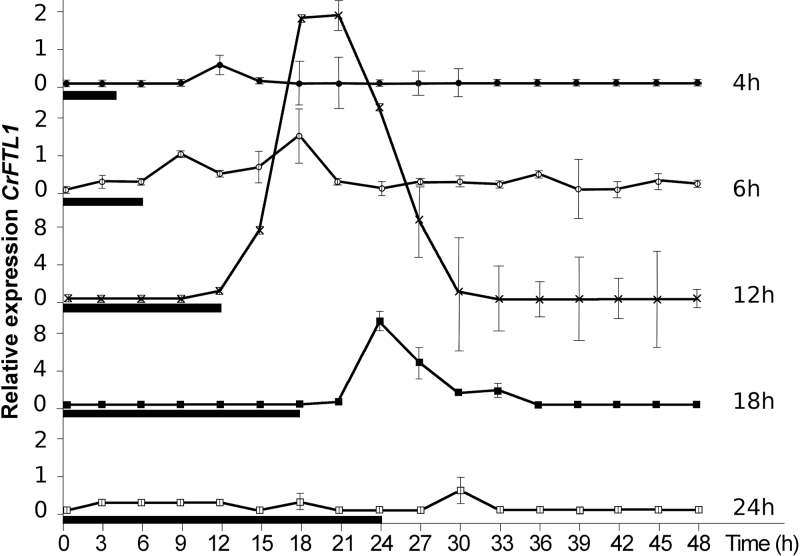
The *CrFTL1* gene is activated after the transfer from dark to light. Five-day-old *C. rubrum* seedlings grown under light were treated by a dark period of various lengths (specified in black boxes). The RNA samples were taken every 3h. Mean values and standard errors (shown as bars) were calculated from four independent measurements of two seedlings (two technical and two biological replicates).

We grew *C. rubrum* seedlings under light for 6 d and then transferred them to dark for 4, 6, 12, 18, or 24h. We measured transcript levels in 3-h intervals (Fig. 1). *CrFTL1* expression was induced by light-on and always reached the maximum 6–8h after a dark–light transition regardless of the extent of preceding darkness. Thus, it was light-on, not previous transition to dark, that determined the timing of the initial peak. *CrFTL1* expression was highest if preceded by a 12-h dark period, lower in the case of 18h of darkness, very low after 6-h and 24-h dark periods, and negligible after 4h of darkness. *CrFTL1* expression correlated with flower induction. The highest proportion of flowering individuals (87%) was found among seedlings exposed to a single 12-h dark period; 33% of plants flowered after 18h of darkness, and no flowering was observed after 4-, 6- or 24-h periods of darkness. We may conclude that *CrFTL1* expression was induced by light-on and its increase reflected the length of the dark period, being maximal after 12h of darkness, which was also the most efficient for flower induction.

### Characterization of *CrCOL* genes

**Fig. 2.**
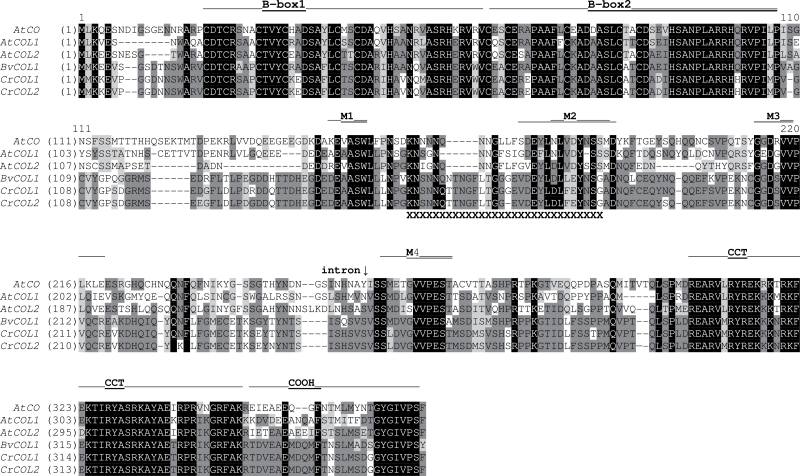
Multiple sequence alignment of CrCOL, BvCOL1, and AtCOL proteins. Conserved domains are marked by the lines above the alignment. Alternatively spliced regions of CrCOL proteins are underlined by XXX. Biochemically similar amino acids are shown on a grey background.

In a search for additional homologues of important flowering-related genes, we identified two full-length clones corresponding to two distinct *CONSTANS-like* genes termed *CrCOL1* and *CrCOL2* in the cDNA library prepared from total RNA of *C. rubrum* ecotype 374. The predicted CrCOL proteins contain two B-boxes, four motifs (M1–M4) in the middle region, CCT and COOH domains (Fig. 2). This structure is specific to Group I, sub-group Ia of COL proteins (Griffiths *et al.*, 2003). *CrCOL1* and *CrCOL2* are highly similar at both nucleotide (97%) and protein (98%) levels. Their closest homologue is *BvCOL1* from sugar beet (Chia *et al.*, 2008), which shows 89% and 90% similarity at nucleotide and protein level, respectively. Just like *BvCOL1*, *CrCOL* genes are closer to *AtCOL2* (67% and 58% similarity at nucleotide and protein level, respectively) than to *AtCO* (63% and 54% similarity at nucleotide and protein level).

**Fig. 3.**
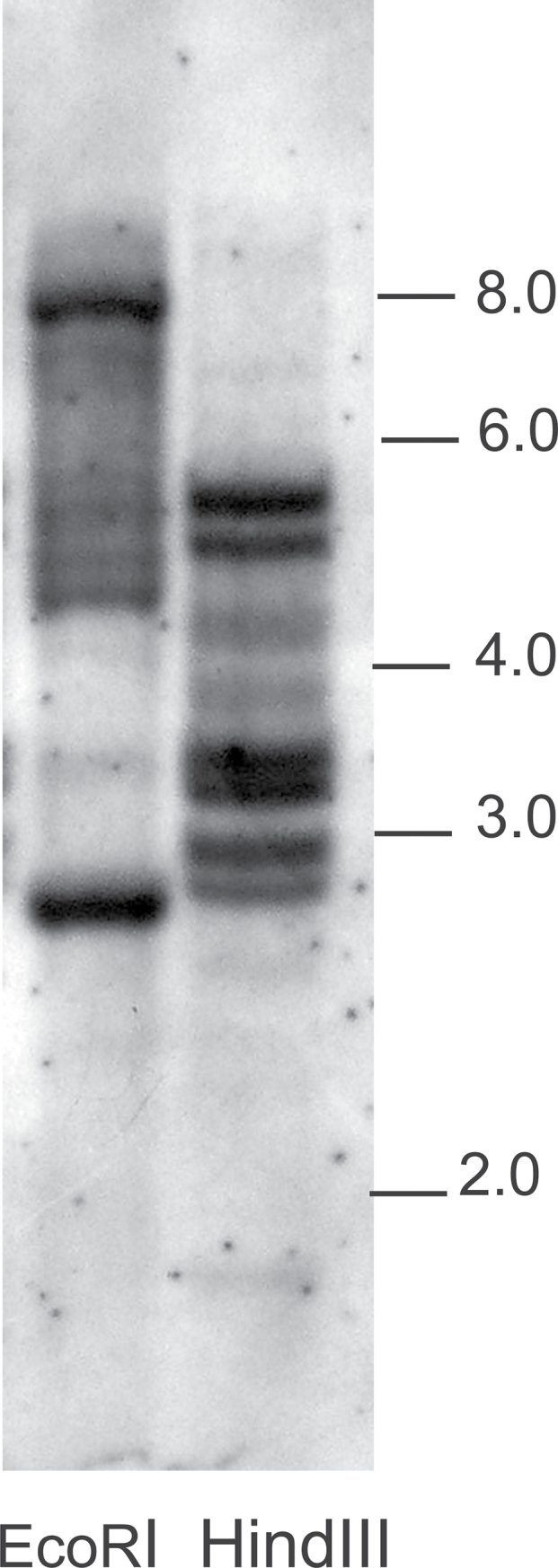
Southern hybridization of *Eco*RI and *Hin*dIII digested DNA with a *COL* probe. Molecular weights in kb are shown on the right.

To estimate the number of *CrCOL* variants, we performed a Southern hybridization with a 0.5-kb probe corresponding to B-boxes and an M1–M2 region under highly stringent conditions. Genomic DNA digested with *Hin*dIII provided six strong and two weak bands, whereas *Eco*RI digest produced two strong and six weaker bands (Fig. 3). As there are no *Hin*dIII or *Eco*RI sites in a probe-covered area in known *COL* genes, two bands in the *Hin*dIII digest should correspond to *CrCOL1* and *CrCOL2*. The remaining four bands are likely derived from two to four additional *COL* variants. The exact number of gene variants depends on whether or not a *Hin*dIII site is present in the area covered by the probe in the sequences of so far unidentified *COL* genes. If *Hin*dIII cleaves the unknown gene copy, two bands instead of one would correspond to one gene. The strong *Eco*RI bands might be derived from two or more restriction fragments of similar sizes, not separated on the gel. We interpret the results of Southern hybridization as the evidence for two to four additional, yet unknown *COL* variants present in a *C. rubrum* genome besides *CrCOL1* and *CrCOL2* genes.

### *CrCOL* genes are alternatively spliced

*CrCOL1* and *CrCOL2* genes possess a single intron located at the same position as in other members of the sub-group Ia *COL* genes. The sizes of the *CrCOL1* and *CrCOL2* introns are 769bp and 752bp, respectively. They are 93% identical, which reflects a close relatedness of both *CrCOL* genes.

In the course of sequencing individual clones from the cDNA library obtained by RACE, we revealed two cDNAs corresponding to *CrCOL* transcripts that were significantly shorter. They were missing 87-bp (or 90-bp) stretches encoding amino acids between positions 152 and 181 (or 182) of CrCOL1 (or CrCOL2) protein (see Fig. 2). The sequence of the missing region, which partially overlapped the M2 domain, started with GT and ended with AG. This suggested the possibility that it might have been an intron spliced out from the primary transcript by alternative splicing. To verify this notion, we performed qRT PCR with primers and probes specific for short transcripts (Supplementary Table S1 available at *JXB* online) with genomic DNA instead of cDNA to reveal a shorter form of the *CrCOL* gene, if it exists. No amplification was observed. In addition, PCR with genomic DNA and the primers targeted to the sequences on both sides of the putative intron produced a single band (not shown). If a shorter form of the *CrCOL* gene was present in *C. rubrum* genome, two bands differing by 90bp would be found. Thus, the transcripts, denoted *CrCOL1s* and *CrCOL2s*, which lack 31 or 30 amino acids, respectively, originated by alternative splicing.

### Overexpression of the *CrCOL* genes in *A. thaliana*

To assess a possible role of the *CrCOL* genes in flower induction we performed a complementation assay of late-flowering *co-2* mutant of *A. thaliana*. We chose one full-length transcript (*CrCOL1*) and one alternatively spliced variant (*CrCOL2s*). The constructs containing particular cDNA under the control of the strong CaMV 35S promoter were transformed into wild type or *co-2* mutants. Between 5 and 15 T1 kanamycin-resistant transformants were obtained for each transgene, and four to five homozygous transgenic lines were selected for detailed analysis. The expression of the transgene was confirmed by qRT PCR measurement. No significant decrease in rosette leaf numbers at time of flowering (Table 1) was observed in transformants compared with recipient plants (wild type or *co-2* mutant) grown under long-day conditions. Three of four lineages *35S::CrCOL1* in *Ler-0* background exhibited a slight but statistically non-significant increase in rosette leaf numbers. The levels of *FT* expression were comparable in all transgenic lineages *35S::CrCOL1*, *35S::CrCOL2* and in wild type. Thus, *CrCOL1* and *CrCOL2s* cannot complement *co* mutation in *A. thaliana* and do not seem to confer flowering suppression.

### The diurnal and circadian pattern of *CrCOL* expression in light and dark

**Fig. 4.**
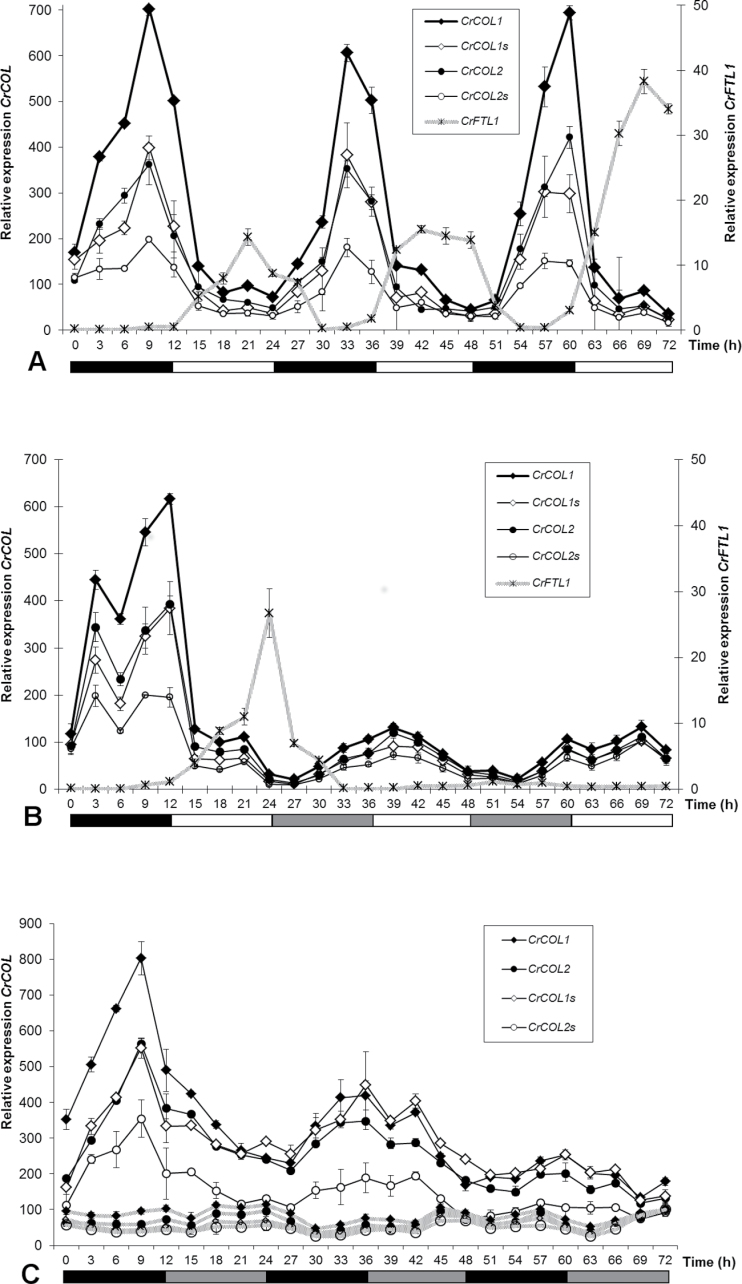
Diurnal and circadian pattern of expression of four splice variants derived from *CrCOL* genes. Five-day-old *C. rubrum* seedlings were grown in the light and then transferred to 12-h dark/12-h light photoperiodic regime (A) or treated by a single period of 12-h darkness followed by constant light (B). Expression of *CrCOL* genes in permanent dark to which 5-day-old seedlings grown under light were transferred is shown in (C). The RNA samples were taken every 3h for 72h. *CrFTL1* also exhibits diurnal rhythmicity, but with opposite amplitudes. Mean values were calculated from two independent measurements of two seedlings (two technical and two biological replicates).

Because we identified four different transcripts derived from two *CrCOL* genes, we wondered how they were expressed. We developed qRT PCR assays specific to each transcript with the aid of TaqMan probes. At first, we examined *CrCOL* expression in *C. rubrum* seedlings grown under the flower-inductive photoperiodic regime of 12h light, 12h dark (Fig. 4A). The expression profiles of the four transcripts were similar. Expression of all *CrCOL* transcripts peaked before dawn and reached minima in the middle of day. A floral inducer *CrFTL1* exhibited diurnal fluctuation, showing peaks of expression anti-phasic to *CrCOL* transcripts, with maxima in the afternoon and minima at night.

To explore free running rhythmicity without continuing entrainment by dark–light cycles, gene expression in *C. rubrum* seedlings grown under permanent light followed by one 12-h dark period was examined (Fig. 4B). The levels of all *CrCOL* transcripts peaked before dawn and showed circadian fluctuation with a period of about 27–30h under permanent light. The main maximum was preceded by a smaller peak at night. A trend toward double peaks was also visible during the first dark period in Fig. 4A. The most prominent feature of *CrCOL* expression under permanent light was its low amplitude. The maxima during light exposure were about five times lower than the peaks achieved in the dark.

Expression of the *CrCOL* genes in *C. rubrum* seedlings grown under constant light for 5 d and then transferred to permanent dark oscillated with a period of about 24–27h (Fig. 4C). The second maximum was about 40% lower than the initial one; a gradual reduction continued across the third peak. However, the decrease was much weaker than under constant light after a 12-h period of darkness (Fig. 4B), when *CrCOL* expression was about five times lower. As in previous experiments, no marked differences were observed among the four transcripts derived from the *CrCOL* genes. Control seedlings grown under permanent light since the initial dark period necessary for germination exhibited very low *CrCOL* expression with slight circadian rhythmicity (Fig. 4C). In conclusion, *CrCOL* expression rhythmically oscillated under constant dark as well as under constant light after entrainment by a single dark period. *CrCOL* transcript levels were always much lower in light than in dark. High levels of *CrCOL* transcripts during darkness were in sharp contrast with *CrFTL1* expression, which also rhythmically oscillated in dark, but with very low amplitudes (Cháb *et al.*, 2008). If plotted onto Fig. 4C, *CrFTL1* expression would follow the zero line. To investigate the effect of light on *CrCOL* gene expression in more detail, their transcript profiles during and after dark periods of different lengths were estimated.

### Light downregulates *CrCOL* transcript levels

**Fig. 5.**
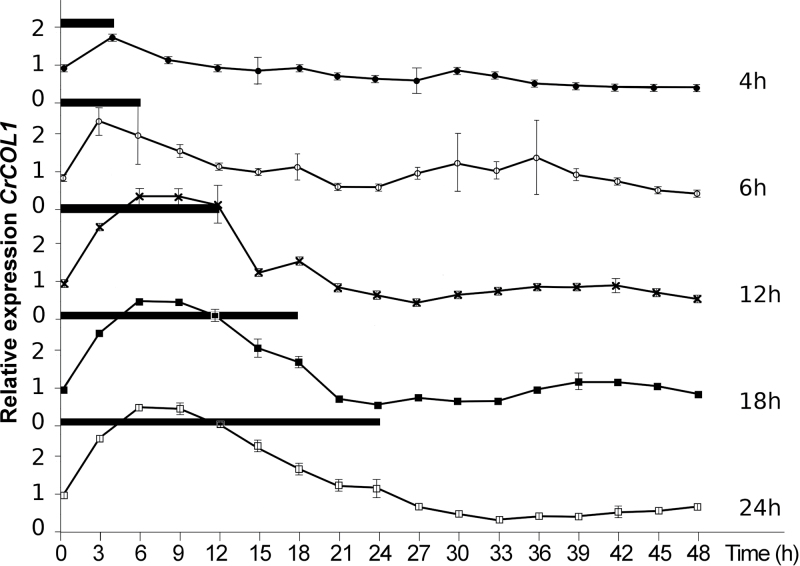
The *CrCOL* genes are downregulated after transfer from dark to light. Five-day-old *C. rubrum* seedlings grown under light were treated by a dark period of various lengths (specified in black boxes). The samples were taken every 3h. All four *CrCOL* splice variants displayed a similar pattern, but only *CrCOL1* is shown for clarity. Mean values and standard errors (showed as bars) were calculated from four independent measurements of two seedlings (two technical and two biological replicates).

*CrCOL* expression was measured in the seedlings during and after a single dark period lasting 4, 6, 12, 18, or 24h, as described previously to examine *CrFTL1* transcripts (see Fig. 1). As all four transcripts showed similar patterns, only *CrCOL1* transcript levels are given in Fig. 5. A dramatic decrease in *CrCOL* expression was observed after light-on. A dark–light transition downregulated *CrCOL* expression regardless of the length of the preceding dark period. Light exhibited the exactly opposite effect on *CrCOL* than on *CrFTL1*, supressing the former and activating the latter.

## Discussion

### A permissive period of darkness followed by light-on is necessary to induce *CrFTL1* expression and flowering in *C. rubrum*

Homologues of the *FT* gene (Kardailsky *et al.*, 1999) have been recognized as floral inducers in many plant species (Carmel-Goren *et al.*, 2003; Hecht *et al.*, 2005; reviewed by Pin and Nilsson, 2012). They are the regulatory targets of *CO*-*like* transcription factors located upstream in a signalling pathway and mediate photoperiod information both in long-day (D’Aloia *et al.*, 2009) and short-day (Hayama *et al.*, 2007) plants. We have previously identified the *CrFTL1* gene, a putative floral promoter in *C. rubrum* (Cháb *et al.*, 2008). In this study, we employed a complementation assay to demostrate that it is indeed a functional equivalent of *FT* in *A. thaliana*. *CrFTL1* is the closest homologue of the sugar beet floral inducer *BvFT2* (Pin *et al.*, 2010), whereas its paralogue *CrFTL2*, which was not capable of complementing *ft* mutant of *A. thaliana*, is closely related to the sugar beet floral repressor *BvFT1* (Pin *et al.*, 2010; Harig *et al.*, 2012).

Rhythmic expression of *CrFTL1* in constant darkness was set up by a light–dark transition (Cháb *et al.*, 2008), analogous to other short-day plants (Kojima *et al.*, 2002; Hayama *et al.*, 2007). However, unlike other short-day plants, the amplitudes were very low or negligible. The high increase in *CrFTL1* transcript level, necessary for flower induction, was always observed several hours after the end of a dark period, regardless of how long it lasted, and thus regardless of the phase of the circadian oscillation at lights-on. In contrast, the duration of a period of darkness determined how pronounced the increase in *CrFTL1* transcript level was. A 12-h dark period which resulted in the highest elevation of *CrFTL1* transcript level also led to the highest proportion of flowering individuals. A recent study of the *GmFT2a* and *GmFT5a* genes in short-day soybean (Kong *et al.*, 2010) revealed diurnal oscillation of their expression peaking in late afternoon, similar to *CrFTL1*. The expression of soybean *FT* homologues in constant darkness was not investigated, hence we cannot determine whether their expression pattern was analogous to *CrFTL1*. Interestingly, both soybean (Buzzell, 1971) and *C. rubrum* (Cummings, 1967) are very efficiently induced to flowering by a 12-h dark period, which is otherwise less efficient for rice or *Pharbitis*. It will be of interest to compare the flower induction pathways of soybean and *C. rubrum* in greater detail.

### *CrCOL* genes are rhythmically expressed and downregulated by light in *C. rubrum*

We identified two novel *COL* genes termed *CrCOL1* and *CrCOL2* with a remarkable expression pattern in a short-day plant *C. rubrum*. They belong to the sub-group Ia, which also contains the gene *CO*, an important element of a photoperiodic regulatory pathway in *A. thaliana*. Expression of *CrCOL1* and *CrCOL2* exhibited diurnal and circadian rhythms, similar to other *COL* genes (Hayama *et al.*, 2007; Almada *et al.*, 2009). However, unlike *CO* in *A. thaliana* (Suarez-Lopez *et al.*, 2001) or *Hd1* in rice (Kojima *et al.*, 2002), it peaked not at the end of the day or at midnight, but rather before dawn. This characteristic resembled the rhythmic expression of *AtCOL1* and *AtCOL2* (Ledger *et al.*, 2001), genes not related to flowering. A similar transcript profile is also specific to the *BvCOL1* gene in sugar beet (Chia *et al.*, 2008), a functional equivalent of *CO*, which represented the closest homologue of *CrCOL* genes found in GenBank (October 2012).

The transition from dark to light dramatically downregulated *CrCOL* transcript levels regardless of the length of a dark period or a phase of rhythmic oscillation. If light persisted after initial darkness, *CrCOL* expression fluctuated with a free running period, but with much lower amplitudes than in the dark. This contrasted with *BvCOL1* in sugar beet, which showed slowly declining expression under constant light (Chia *et al.*, 2008). Light positively influences *CO* transcription in *A. thaliana* under long days and is responsible for its biphasic expression pattern, with an additional peak at dusk (Suarez-Lopez *et al.*, 2001). A higher level of *CO* mRNA and a higher stability of CO protein under long days (Valverde *et al.*, 2004) leads to *FT* activation and then flower induction. Thus, *CO* expression transmits information about day length. It is possible that downregulation of the *CrCOL* genes by a dark–light transition contributes to the perception of night length in short-day *C. rubrum*.

The rhythmic expression pattern of the *CrCOL* genes suggested that they may be involved in perceiving dark periods and thus in control of processes dependent on day–night cycles, e.g. flower induction. However, the examined *CrCOL* genes did not complement *co* mutants of *A. thaliana*, unlike *BvCOL1* (Chia *et al.*, 2008) and other *COL* homologues (Liu *et al.*, 2001). A negative result of heterologous complementation cannot exclude the possibility that the *CrCOL* genes were involved in flowering control in *C. rubrum*, but could not function properly in *A. thaliana*. Overexpression and silencing of the C*rCOL* genes in *C. rubrum* will provide more conclusive evidence about the function of these genes.

### Numerous *CrCOL* transcripts in *C. rubrum*

Alternative splicing generated shorter transcripts derived from the *CrCOL1* and *CrCOL2* genes that lacked a region encoding a part of the M2 domain. The examination of expression of *CrCOL* splice variants under various photoperiodic regimes in *C. rubrum* seedlings did not provide a clue about a possible role of this phenomenon. All *CrCOL* splice variants displayed very similar expression profiles. The M2 domain, affected by alternative splicing, is one of four evolutionary conserved motifs located between the B-boxes and the CCT region. Its function is not known, but some segments in this area, particularly glutamine-rich sequences, play a role in transcriptional activation and are capable of binding to DNA (Tiwari *et al.*, 2010). It is possible that alternative splicing may influence the choice of the genes controlled by CrCOL factors.

Alternative splicing contributes to the notable richness of *COL* transcripts in *C. rubrum*. Two to four additional *COL* genes, highly similar to known *CrCOL* genes, were revealed by Southern hybridization. This contrasts with only one sub-group Ia gene, *BvCOL1*, found by means of Southern hybridization in sugar beet by Chia *et al.* (2008). The larger number of *COL* genes in *C. rubrum* may reflect their functional diversification. *COL* genes in higher plants are involved in the control of various developmental processes, not only flower induction (Datta *et al.*, 2006; Almada *et al.*, 2009) but also in response to abiotic stresses (Winter *et al.*, 2007). *Chenopodium rubrum* is tolerant to relatively high salt and nitrate concentrations (Wisskirchen, 2006). Some members of the numerous *COL* transcripts may help the plant to cope with osmotic or drought stresses.

*CrCOL1* and *CrCOL2* are 97% identical and share similar introns. They may therefore represent homeologs originating from diploid parents of tetraploid *C. rubrum*. Polyploidy certainly contributed to increased number of *CrCOL* genes. To decipher the roles of individual members of this family, complementation assays in *A. thaliana* are helpful. However, silencing or overexpression of the genes under study in *C. rubrum* (Veit *et al.*, 2006) will be necessary to reveal their functions.

### *CrCOL* and *CrFTL1* expression profiles show complementary expression levels

The comparison of *CrCOL* and *CrFTL1* expression in *C. rubrum* seedlings revealed an interesting juxtaposition: *CrCOL* maxima corresponded to *CrFTL1* minima in diurnal oscillation of transcript levels and vice versa. Also, when the *CrCOL* genes were downregulated by light-on after various periods of darkness (Fig. 5), *CrFTL1* was upregulated. If *CrCOL* genes were involved in flowering control, they would play the role of a suppressor rather than a promoter.

The investigation of *FT* and *COL* homologues in *C. rubrum* uncovered noteworthy features of their expression not reported in other short-day plants. Expression of the floral promoter *CrFTL1* was induced by light-on and depended on the length of a preceding dark period. Expression of the *CrCOL* genes exhibited diurnal and circadian rhythmicity and also light sensitivity, suggesting their role in the processes is controlled by day–night cycles, although more research is needed to understand their function. Thus the short-day plant *C. rubrum* deserves scientific attention as a suitable model for comparison with the long-day sugar beet in the same family, Amaranthaceae.

## Supplementary material

Supplementary data are available at *JXB* online.

Table S1. The primers and probes.

Supplementary Data

Supplementary Data
