# Supplementary material for: A dark–light transition triggers expression of the floral promoter CrFTL1 and downregulates CONSTANS-like genes in a short-day plant Chenopodium rubrum
Source: J Exp Bot. 2014 Mar 18;65(8):2137–46. doi: 10.1093/jxb/eru073 (PMC3991744; doi:10.1093/jxb/eru073)
Supplement: Supplementary Data [file supp_eru073_jexbot118026_file001.pdf]

Table S1. Primers and probes used to clone, to sequence or to amplify *CONSTANS* like and *FT* like genes or transcripts in *C. rubrum*. Sequences are shown for the oligonucleotides designed in this study.

Primer purpose	Primer name	Sequence (5'> 3') or reference
***CrCOL* universal**	CONDEG94F	GAGCTAATCCTCTTGCTAGAAGACAYCANMGNRT
	CONDEG307R	TTTTCTAGTTTTCTTCTTTTCTCTATATCTAAGAACNCKNGCYTC
	CrCON_5UTR	GGGACAGCAGAGAGAACCAA
	CrCON_3UTR	TTTATTGGCAGCACAACCAA
**RACE**	CrCON1523rev	GGCCTTGAATGTGACACAGA
	CrCON138for	GTAAGGGGATGAGGATGAAG
	CrCON1456for	TCCTCCCTAGATGTGGGTGT
	CrCON1508for	TGTCACATTCAAGGCCTCCT
***CrCOL* Southern**	COL_F1	GTTTGTGACACATGCCGTTC
	COL_R1	TGCTGCTGGTTATACTGCTC
***CrCOL* intron**	CrCOL1intrFOR	TTTTGTTCGGGATGGAATGT
	CrCOL1intrREV	TGAGTTGGAGATAAACCCCAGT
	CrCOL2intrF	TTTTGTTCGGAATGGAATGT
	CrCOL2intrR	CACCCACATCTAGGGAGGAG
***CrFTL* Cloning**	CrFTL1for	CAGGCAGATATATAGAGTTGTCGAA
	CrFTL1rev	TCCAATGATCTCTTCACGGTAG
	CrFTL2f	GGGGACAAAACTTCCAATGA
	CrFTL2r	GACATTAGGCAAACACTTTGAGAA
**qRT PCR**		
CrCOL1	252&51F	GGATTCCTGGACCTACCAGACA
	252&54R2	TCTGCACCTGAATTATACTCAAAGAG
	TM252	**FAM**-CCTCCTCCAGTTAAAAACCCATTT-**TMR**
CrCOL1s	252&54R1	CAGAAYTGATTATCTGCACCTGGG
	TMall	**FAM**-CGAAGCAGCTTCATCCTCATCCCC-**TMR**
	252&51F	This study
CrCOL2	54&256F	GATTCCTGGACCTGCCAGATG
	252&54R2 and TMall	This study, see above
CrCOL2s	54&256F, 252&54R1, TMall	This study, see above
CrFTL1	CrFT720-298Rev	Cháb et al. 2008
	CrFT720-52For	
CrFTL2	CrFT787-361For	Cháb et al. 2008
	CrFT787-536Rev	
actin	ACT-2_for	Cháb et al. 2008
	ACT-2_rev	
