# Supplementary material for: A dark–light transition triggers expression of the floral promoter CrFTL1 and downregulates CONSTANS-like genes in a short-day plant Chenopodium rubrum
Source: J Exp Bot. 2014 Mar 18;65(8):2137–46. doi: 10.1093/jxb/eru073 (PMC3991744; doi:10.1093/jxb/eru073)
Supplement: Supplementary Data [file supp_65_8_2137_v2_index.html]

A dark–light transition triggers expression of the floral promoter CrFTL1 and downregulates CONSTANS-like genes in a short-day plant Chenopodium rubrum — Supplementary Data 

# A dark–light transition triggers expression of the floral promoter *CrFTL1* and downregulates *CONSTANS-like* genes in a short-day plant *Chenopodium rubrum*

## Supplementary Data

Data files

**Files in this Data Supplement:**

- Supplementary Data - Supplementary Data
